# Spondyloenchondrodysplasia in five new patients: identification of three novel *ACP5* variants with variable neurological presentations

**DOI:** 10.1007/s00438-023-02009-1

**Published:** 2023-04-03

**Authors:** Rasha M. Elhossini, Hasnaa M. Elbendary, Karima Rafat, Raghda M. Ghorab, Mohamed S. Abdel-Hamid

**Affiliations:** 1 Clinical Genetics Department, Institute of Human Genetics and Genome Research, National Research Centre, Cairo, Egypt; 2 Immunogenetics Department, Human Genetics and Genome Research Institute, National Research Centre, El-Bohous Street, El-Dokki, Cairo, 12622 Egypt; 3 Medical Molecular Genetics Department, Institute of Human Genetics and Genome Research, National Research Centre, Cairo, Egypt

**Keywords:** Spondyloenchondrodysplasia, SPENCD, Growth hormone, Intracranial calcifications, Immune dysregulation, *ACP5* gene

## Abstract

Spondyloenchondrodysplasia (SPENCD) is an immune-osseous disorder caused by biallelic variants in *ACP5* gene and is less commonly associated with neurological abnormalities such as global developmental delay, spasticity and seizures. Herein, we describe five new patients from four unrelated Egyptian families with complex clinical presentations including predominant neurological presentations masking the skeletal and immunological manifestations. All our patients had spasticity with variable associations of motor and mental delay or epilepsy. All except for one patient had bilateral calcification in the basal ganglia. One patient had an associated growth hormone deficiency with fair response to growth hormone therapy (GH) where the height improved from −3.0 SD before GH therapy to −2.35 SD at presentation. Patients had different forms of immune dysregulation. All patients except for one had either cellular immunodeficiency (3 patients) or combined immunodeficiency (1 patient). Whole exome sequencing was performed and revealed four *ACP5* variants: c.629C > T (p.Ser210Phe), c.526C > T (p.Arg176Ter), c.742dupC (p.Gln248ProfsTer3) and c.775G > A (p.Gly259Arg). Of them, three variants were not described before. Our study reinforces the striking phenotypic variability associated with SPENCD and expands the mutational spectrum of this rare disorder. Further, it documents the positive response to growth hormone therapy in the studied patient.

**Supplementary Information:**

The online version contains supplementary material available at 10.1007/s00438-023-02009-1.

## Introduction

In this study, we describe five new patients with Spondyloenchondrodysplasia presenting with severe neurological manifestations and mild skeletal and immunological changes. Such variable phenotypic presentations were masking the disease and led to the delay in diagnosis until exome sequencing was used revealing homozygous variants in the *ACP5* gene as the cause of the patients’ phenotype. In addition, we present the beneficial use of growth hormone therapy in one patient.

Spondyloenchondrodysplasia (SPENCD, OMIM# 271550) is a very rare autosomal recessive spondylometaphyseal dysplasia characterized by variable degrees of metaphyseal changes, enchondromas, platyspondyly and short stature. It is commonly associated with immune dysregulation such as immune deficiency or autoimmune diseases, in addition to neurological impairment in the form of global developmental delay, spasticity, seizures, ataxia, leukodystrophy and intracranial calcifications (Girschick et al. 2015; Gelen et al. 2021). Briggs et al 2016 proposed that SPENCD with immune dysregulation (SPENCDI, OMIM: 607944) and SPENCD (OMIM: 271550) represented a continuum of the same disorder. However, SPENCD (OMIM: 271550) has already been incorporated into SPENCDI (OMIM: 607944).

SPENCD can be differentiated from other spondylometaphyseal dysplasias by the presence of bone enchondromas which are radiographic radiolucent spondylar and metaphyseal lesions caused by persistence of chondroid tissue within the ossified bones (Renella et al. 2006). It has a variable onset, severity and clinical presentations in addition to the presence of inter-familial variability (Navarro et al. 2008). However, Zhong et al. (2018) reported that skeletal abnormalities are the most common presentation followed by autoimmune diseases and central nervous system involvement.

SPENCD is caused by biallelic mutations in *ACP5* gene, located on 19p13.2 that encodes tartrate-resistant acid phosphatase (TRAP) (Briggs et al. 2011; Lausch et al. 2011). *ACP5* mutations cause loss of regulation of TRAP activity on osteopontin function (OPN). The functional excess of phosphorylated OPN causes an increase in bone resorption by the activation of osteoclasts. In addition, it causes immune dysregulation by the stimulation of type I interferon production (Wong et al. 2005; D’Alfonso et al. 2005). Elevation of type I interferon is associated with many autoinflammatory disorders known as “Type I interferonopathies” including Aicardi-Goutières syndrome (type 1–7), STING-associated vasculopathy, infantile-onset, X-linked reticulate pigmentary disorder, USP 18 deficiency, chronic atypical neutrophilic dermatitis with lipodystrophy, Singleton–Merten syndrome and ADA2 deficiency, as well as SPENCD (Crow 2015; Volpi et al. 2016; Picard et al. 2018; Yu and Song 2020). Recently, Type I interferonopathies were updated by the 2022 classification of International Union of Immunological Societies Expert Committee (IUIS) to include 6 new disorders (Tangye et al. 2022).

## Materials and methods

### Patients

The study included five Egyptian patients from four unrelated families with the clinical and radiological findings of SPENCD. Written informed consents were obtained from the patients’ parents for publication of this report and any accompanying images. The data supporting the findings of this study are available with the corresponding author upon request. This study was approved by the Medical Research Ethics Committee at National Research Centre, Cairo, Egypt.

### Methods

Complete clinical evaluation, extended pedigree analysis, neuro-imaging, skeletal survey, immunological profile, and endocrinal assessment were done for all affected. Parents were evaluated for any minor features.

### Immunological investigations

Lymphocyte subsets’ immunophenotyping was done using flow cytometry (BD Accuri™ C6 Cytometer, USA). Analysis of lymphocyte surface markers was done on lysed whole blood by BD FACS™ lysing solution using CD3 FITC labeled monoclonal antibodies for T lymphocytes, CD16 labeled with PE for Natural Killer (NK) cells and FITC labeled CD19 for B lymphocytes. CD4 PE and CD8 FITC (BD Biosciences, USA) were used for further quantitation of T helper and T cytotoxic lymphocytes populations, respectively. Immunoglobulin levels in serum were measured by immunoturbidimetry.

### Whole exome sequencing

Genomic DNA was extracted from peripheral blood samples of patients and their parents using Qiagen Blood DNA Kit (Qiagen, Hilden, Germany). A solo whole exome sequencing was performed for one patient from each family using SureSelect Human All Exome 50 Mb Kit (Agilent, Santa Clara, CA, USA) and analyzed on Illumina NovaSeq 6000 (Illumina, San Diego, CA, USA). The obtained sequences were aligned to UCSC human genome GRCh37/hg19 and variants were verified through the GATK pipeline. Annotation of variants was done using BaseSpace Variant Interpreter Server. Identified variants were checked against public genetic databases like Genome Aggregation Database (gnomAD, https://gnomad.broadinstitute.org/), 1000 Genomes (www.1000genomes.org), and dbSNP (http://www.ncbi.nlm.nih.gov/SNP/). Pathogenicity of detected missense and splice site variants were predicted using various bioinformatics tools as SIFT (https://provean.jcvi.org/protein), PolyPhen-2 (https://genetics.bwh.harvard.edu/pph2/) and MutationTaster (https://www.mutationtaster.org/).

### Segregation analysis

Variants identified by exome sequence were further confirmed in the parents and other available family members using Sanger sequencing. Exons harboring the variants were amplified using specific primers designed by ExonPrimer SOFTWARE. Primers are available upon request from the corresponding author. PCR cycling conditions were: initial denaturation at 96 °C for 5 min; 30 cycles of denaturation at 96 °C for 30 s; annealing at 60 °C for 30 s; extension at 72 °C for 30 min, and a final extension at 72 °C for 5 min. PCR products were purified using Exo-SAP PCR Clean-up kit (Fermentas, Germany) and sequenced in both directions using the BigDye Terminator v3.1 Cycle Sequencing Kit (Applied Biosystems, Foster City, CA, USA) and analyzed on the ABI Prism 3500 Genetic Analyzer (Applied Biosystems) according to the manufacturer's instructions.

## Results

The detailed clinical and molecular data of our patients are presented in Table 1.

**Table 1:** The clinical, immunological and molecular characteristics of our patients with SPENCD

Items	Patient 1	Patient 2	Patient 3	Patient 4	Patient 5
Age	5y	3y 10 m	12y	4y 3 m	2y 8 m
Gender	F	F	M	M	F
Consanguinity	+	+	+	+	+
Similarly affected family member	+	+ (cousin of Patient 1)	−	−	−
Weight kg (SD)	10 (− 4.08SD)	9 (− 4.08SD)	30 (− 1.77SD)	15 (− 1.08SD)	11 (− 1.76SD)
Height cm (SD)	88 (− 4.31SD)	78 (− 5.24SD)	132 (− 2.35SD)	98 (1.4SD)	82 (− 2.6SD)
OFC cm (SD)	49 (− 0.94SD)	47 (− 1.59SD)	52 (− 1.21SD)	50 (− 0.42SD)	46.5 (− 1.15SD)
Disease onset	1y 2 m	1y 8 m	2y	4 m	8 m
Regression	+	+	−	+	+
Motor delay/milestones	+ /Walked support at 1y 3 m but frequent falling	+ /Never achieved only sat unsupported at 10 m	−/1 year and 3 m	+ /Head support at 6 m, cannot sit or walk	+ /Head support at 6 m. sat at 1y, cannot stand or walk
Gait	Cannot walk	Cannot walk	Abnormal unsteady gait	Cannot walk	Cannot walk
Cognitive delay	+	+	−	+	−
1st spoke	12 m	13 m	12 m	−	vocalized
Speech	Few words not clear	Now absent speech	Normal speech	Absent speech	Two words sentences
Hearing	N	N	N	N	N
Vision	N	N	N	N	N
Facies	Dolichocephaly, high anterior hairline, frontal bossing, long philtrum	Dolichocephaly, high anterior hairline, frontal bossing, long philtrum, large simple ears	Dolichocephaly, high anterior hairline, frontal bossing, long philtrum, large simple ears	Brachycephaly, high anterior hairline, short philtrum, cupped, low set posteriorly rotated ears	Not dysmorphic
Spasticity	Spastic quadriparesis	Marked spastic quadriparesis	No spasticity	Spastic paraplegia, could use hands	Spastic paraplegia, could use hands
Deep tendon reflex	Brisk	Brisk	Brisk	Brisk	Brisk
Previous surgical procedures	−	−	Tendon release twice at 4 y and 9 y	Huge omphalocele (performed at 3 days old)	−
Fits	−	−	+	Only sleep disturbances	−
Onset of fits	−	−	6 m	−	−
Type of fits	−	−	Absence	−	−
Frequency of fits	−	−	Every couple of months	−	−
Drug response	−	−	Fairly controlled on Na Valproate	−	−
EEG	N	N	Epileptogenic activity bi− temporal and occipital discharge	Epileptogenic activity At left centrotemporal area	N
Brain CT Scan	Bilateral calcifications in basal ganglia	N/A	Bilateral calcifications in basal ganglia and in frontal lobe	Bilateral calcifications in basal ganglia	Bilateral calcifications in basal ganglia
Brain MRI	Thin CC, dilated asymmetrical lateral ventricle, deep white matter changes	Thin CC	N/A	Minimal cortical increased involution, dilated lateral ventricle, bilateral deep white matter signal at T2 around the occipital area	Thin CC
Broad joints	Mild	Mild	Notable	Mild	Mild
Short stature	+	+	+	−	+
GH deficiency	−	−	+	−	−
Platyspondyly	+	+	+	+	+
Metaphyseal dysplasia	+	+	+	+	+
Metaphyseal enchondromatosis	−	−	+	−	+
Bone age	Delayed	Delayed	Delayed	Delayed	N
EMG, NCV	N	N	N	N	N
CPK	N	N	N	N	NA
Skin and mucosa involvement	−	Recurrent skin rash	−	−	Eczema
Hematological manifestations CBC HB RBCs shape Platelets WBCs	Anemia Anisopoikilocytosis thrombocytopenia N	Anemia Anisocytosis N N	N N N Neutropenia	N N N Absolute lymphopenia and monocytopenia	Anemia N N Relative lymphocytosis
Immunological manifestations Infections	−	Recurrent skin rash	−	Frequent attacks of fever Chest infections	Neonatal fever Eczema Food allergy; specifically eggs
Cellular immunity CD3 T lymphocytes CD4 T helper CD8 T cytotoxic CD4/CD8 Ratio CD19 B lymphocytes CD16 NK cells	Decreased (R) Decreased (R&A) N Reversed N N	Decreased (R&A) Decreased (R) Decreased (R&A) N N N	Decreased (R&A) Decreased (R&A) Decreased (R) Reversed N N	Decreased (R) Decreased (R&A) Decreased (A) Reversed Decreased (A) Decreased (A)	Increased (A) Increased (A) N N N N
Humoral Immunity IgG IgM IgA IgE	Elevated IgG Normal IgM Elevated IgA NA	Elevated IgG Normal IgM Normal IgA NA	Elevated IgG Normal IgM Elevated IgA NA	Normal IgG Normal IgM Low IgA NA	Normal IgG Normal IgM Elevated IgA Elevated IgE
*ACP5* variant (NM_001111035.2)	c.629C > T p.(Ser210Phe)	c.629C > T p.(Ser210Phe)	c.526C > T p.(Arg176Ter)	c.742dupC p.(Gln248ProfsTer3)	c.775G > A p.(Gly259Arg)
Classification of variants according to ACMG	VUS	VUS	Likely Pathogenic	Likely Pathogenic	VUS

*A* absolute count, *ACMG* American College of Medical Genetics, *CBC* complete blood count, *CC* corpus callosum, *CPK* creatine phosphokinase, *EEG* electroencephalogram, *EMG and NCV* electromyography and nerve conduction velocity, *F* female, *GH* growth hormone, *HB* Hemoglobin, *Ht* height, *LL* lower limbs, *M* male, m month, *N/A* not available, *NK cells* natural killer cells, *NN* normocytic normochromic, *OFC* occipitofrontal circumference, *N* normal, *R* relative count, *RBCs* red blood cell, *SD* standard deviation, *VUS* variant of uncertain significant, *WBCs* white blood cells, *Wt* weight, *y* years

### Clinical data

**Fig. 1:**
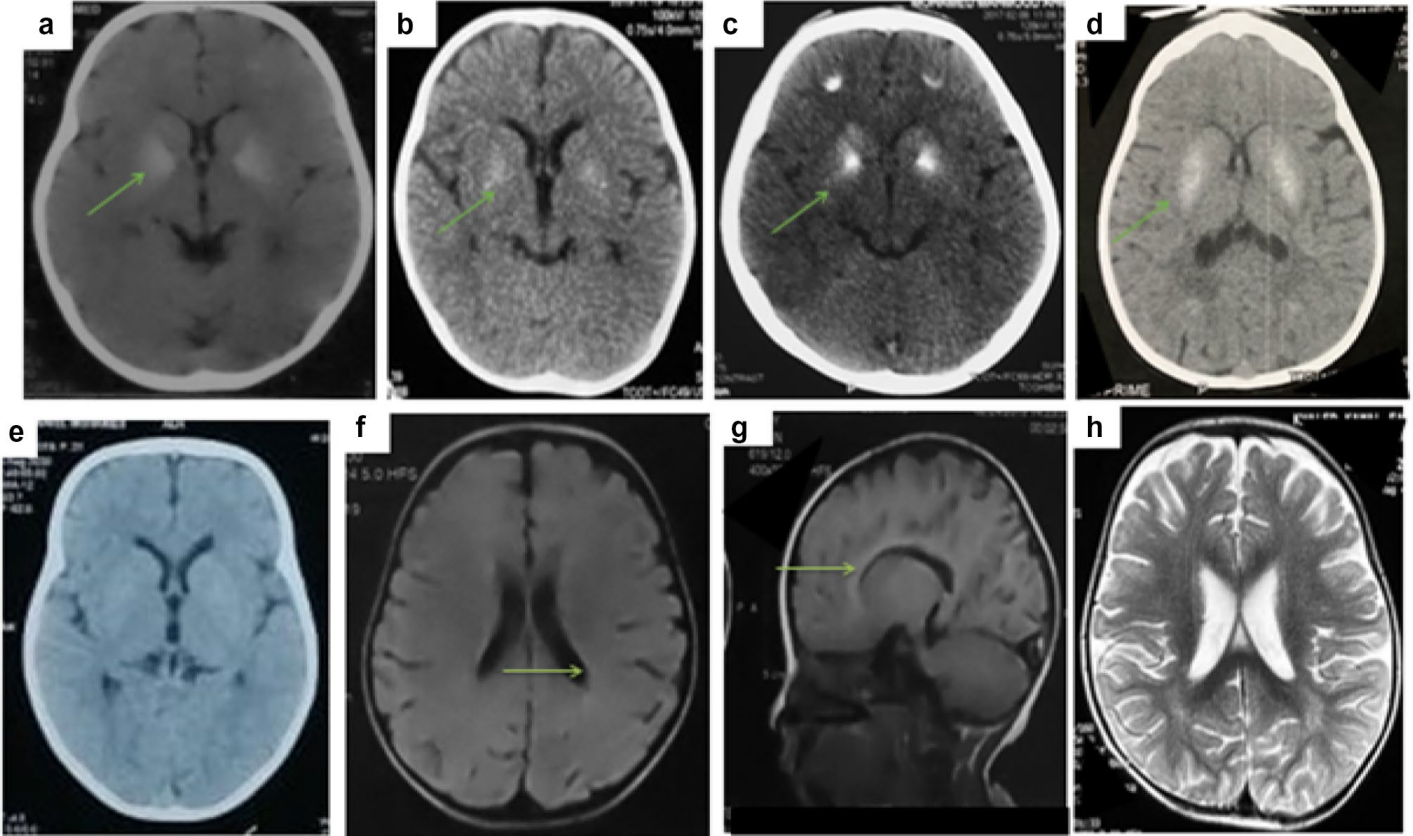
**a**–**d** Brain CT scans showing calcification in basal ganglia in the Patients 1, 3, 4 and 5/ respectively. **e** Brain CT showing absence of calcifications in Patient 2. **f**, **g** Brain MRI of Patient 2 showing asymmetry in lateral ventricles and thin corpus callosum. **h** Brain axial (T2) MRI in Patient 4 showing mild deep white matter signal and minimal cortical and central atrophic changes

**Fig. 2:**
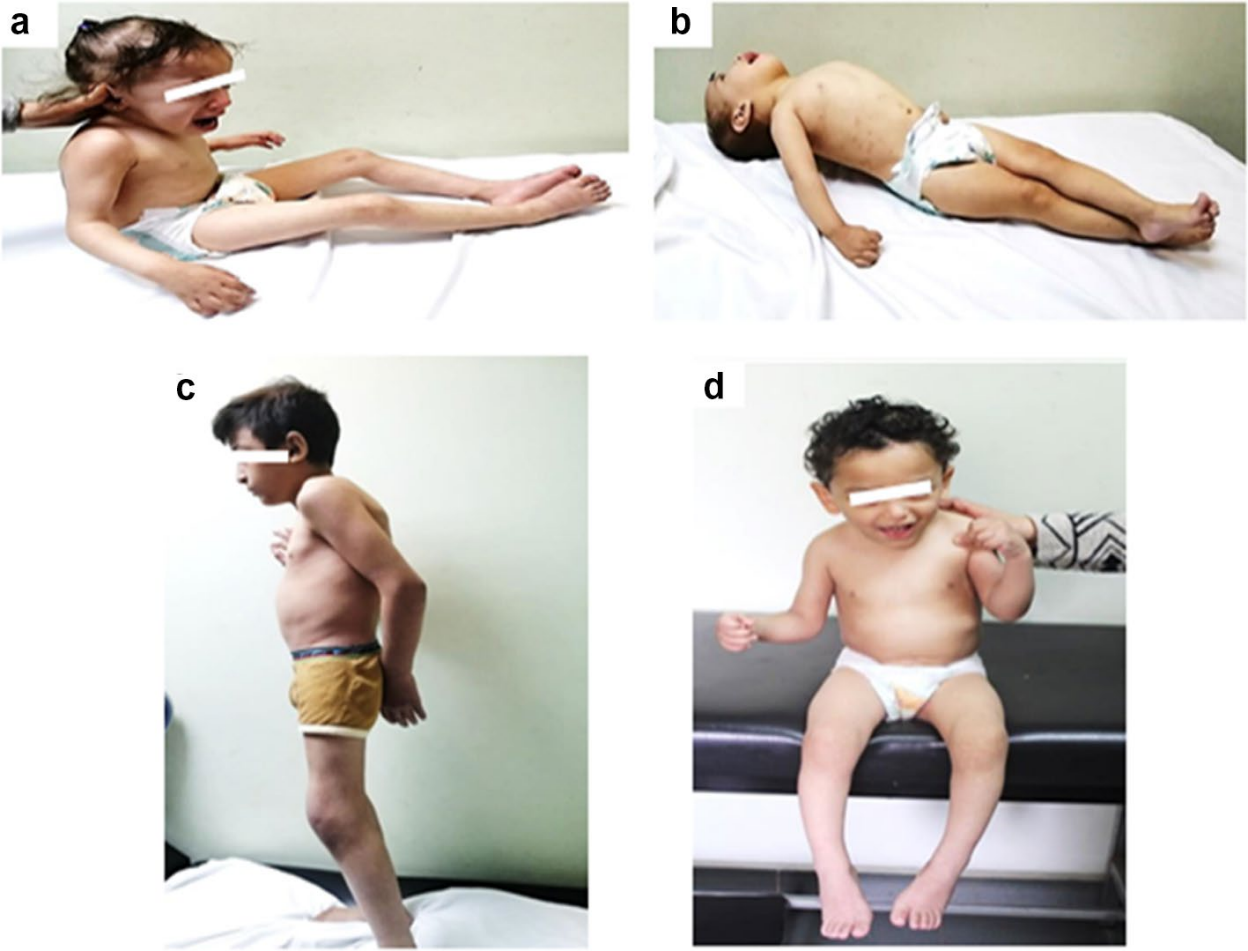
**a**, **b** Patients 1 and 2 at the age of 5 years and 3 years and 10 months, respectively. Note spasticity specially in Patient 2. **c** Patient 3 at the age of 12 years. Note limited knees extension. **d** Patient 4 at the age of 4 years and 3 months with minimal knees’ broadening

**Fig. 3:**
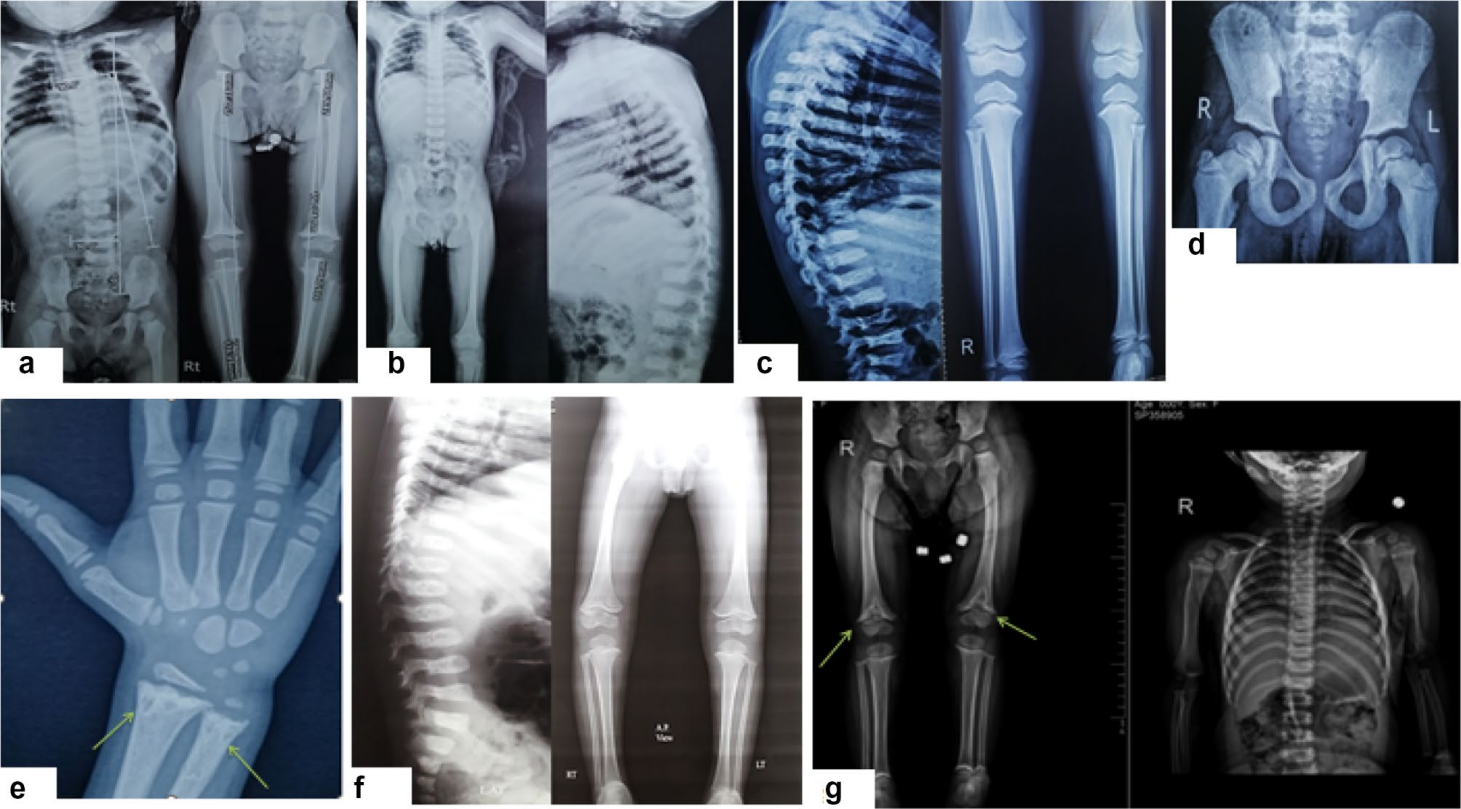
**a**, **b** Skeletal survey of Patients 1 and 2 showing platyspondyly, flat acetabulum, short broad femoral necks and broad sclerotic metaphysis, in addition to mild scoliosis in Patient 1. **c**–**e** Skeletal survey of Patient 3 at 12 years old (6 years with GH therapy) showing platyspondyly, flat acetabulum, short broad femoral necks, broad sclerotic metaphysis and delayed bone age in addition to thoracic kyphosis and metaphyseal enchondromas at proximal end of right fibula and distal ends of left radius (arrows). **f** Skeletal survey of Patient 4 showing platyspondyly, flat acetabulum, short broad femoral necks and broad sclerotic metaphysis. **g** Skeletal survey of Patient 5 at 2 years and 8 months revealed platyspondyly, flat acetabulum, short broad femoral necks, coxa valga, and broad sclerotic metaphysis in addition to bilateral metaphyseal enchondromas at distal ends of femurs (arrows)

Our patients were three females and two males. Their ages ranged from 2 years and 8 months to 12 years. Parental consanguinity was positive in all of them. Family history of a similarly affected family member was observed in one family (Family 1, Patient 1 and 2). In all patients, gestational and delivery histories were unremarkable. Disease onset was presented at infancy with progressive loss of acquired motor developmental milestones and progressive spasticity in Patients 1, 2, 4 and 5. Patient 3 had a history of normal motor and mental developmental milestones with progressive spasticity and gait abnormalities noticed at age of 2 years. Patients 1, 2 and 4 had an associated intellectual disability; however, the participating patients did not undergo tests for cognitive evaluation. Electroencephalogram was normal in three patients (Patients 1, 2 and 5). Patient 3 had a history of two attacks of febrile convulsion then experienced repeated attacks of absence seizures since the age of 7 years that were fairly controlled on medications. Patient 4 had sleep disturbances, but no documented attacks of epilepsy although EEG showed generalized epileptogenic focus. Brain imaging was done and showed intracranial calcification in four patients, thin corpus callosum in three patients, dilated lateral ventricles and deep white matter changes in two patients (Fig. 1). Patients 1 and 3 neither had apparent infections, recurrent fevers, skin, joint problems nor chronic gastrointestinal complaints as warning signs for immunodeficiency or autoimmune diseases. Patient 2 had a few attacks of skin rash. Patient 4 experienced a few attacks of fever and chest infections that required hospital admission twice and patient 5 had a history of eczema and food allergy (eggs). All patients had minimal broadening of joints that was more apparent in Patient 3 (Fig. 2). All patients except Patient 4 had an associated short stature (height was between −5.24 SD and −2.35 SD). Growth hormone (GH) level basal and after stimulation by clonidine and insulin was normal in Patient 1, 2, 4 and 5 and deficient in Patient 3 who had received GH replacement for 6 years with fair response. Skeletal survey of all patients showed platyspondyly, flat acetabulum, short broad femoral necks, broad sclerotic metaphysis and delayed bone age (Fig. 3). Neurophysiologic examination of muscles and nerves was normal in all of them as well as CPK, serum calcium, phosphorus, alkaline phosphatase, parathyroid hormone and Vitamin D3 were normal.

### Molecular data

**Fig. 4:**
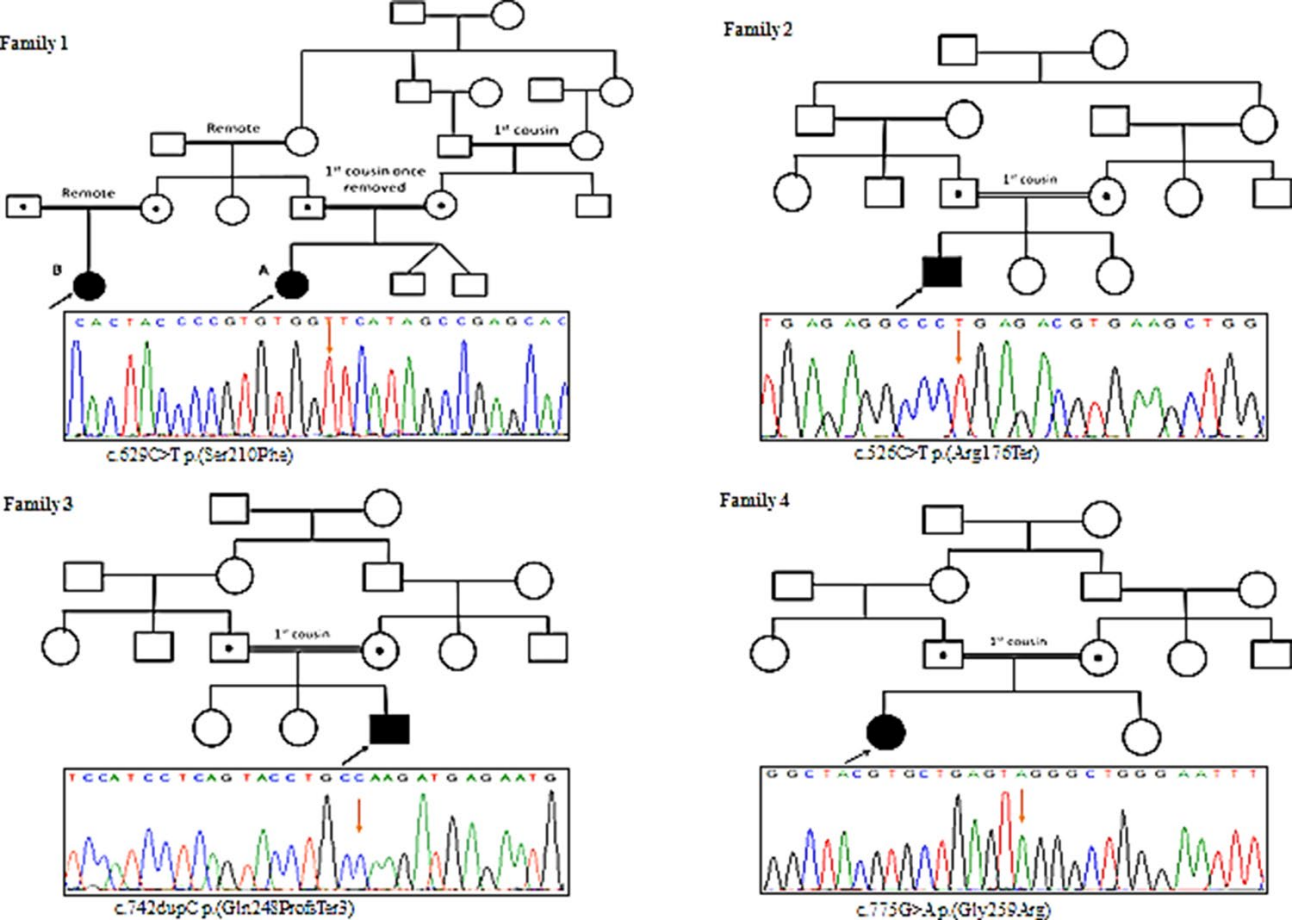
Pedigrees of the four studied families and portions of the sequencing electropherograms showing the different *ACP5* variants identified. Arrow indicates site of mutation

Exome sequencing was performed for one patient from each family. Thorough investigations and filtering of the exome data revealed homozygous variants in the *ACP5* gene in all our patients. No other variants (pathogenic, likely pathogenic, or variants of uncertain significance) that can be relevant to the patients’ phenotype were found. Overall, four different *ACP5* variants were identified: c.526C > T (p.Arg176Ter), c.629C > T (p.Ser210Phe), c.742dupC (p.Gln248ProfsTer3), and c.775G > A (p.Gly259Arg) (Fig. 4). All except the c.526C > T (p.Arg176Ter) were not described before. Variants segregated perfectly with the phenotype in all families, being heterozygous in the respective parents and either wild type or heterozygous in unaffected healthy sibs. The three novel variants were absent in public genetic databases and our in-house database of more than 1000 exome of Egyptian origin. Further, they were predicted to be deleterious by various bioinformatic tools (Supplementary Table 1).

## Discussion

SPENCD is a rare complex recessive skeletal dysplasia characterized by platyspondyly, enchondroma-like radiolucent metaphyseal lesions, metaphyseal dysplasia of the long bones, and commonly short-trunked short stature in addition to variable extra-skeletal presentations (Utsumi et al. 2017).

Briggs et al. (2016) noted the marked clinical variability in SPENCD, within and between families with age at onset ranging from birth to 15 years and severity ranging from death at infancy due to autoimmune thrombocytopenia to isolated skeletal dysplasia in a 36-year-old patient. The disease variability and severity were obviously shown in our five patients. Although skeletal findings were described as the main diagnostic clue in SPENCD, they were minimal in Patients 1 and 2 in the form of mild joints’ broadening and almost unnoticed in Patient 4 and 5. Only Patient 3 has notable broadening of joints that was misdiagnosed as nutritional rickets and was initially treated with calcium supplementation only. This highlights the importance of performing skeletal survey in patients with nutritional rickets in association with short stature as it might have a diagnostic benefit as reported by Flechtner et al. (2014). In our study, it is noteworthy that the reason for seeking medical advice was the neurological impairment in the form of early onset spasticity in all of them (100%), and significant developmental delay in Patients 1, 2, 4 and 5 (80%). This is in contrast to the studies of Utsumi et al. (2017) and Zhong et al. (2018) as they both reported that the musculoskeletal presentation was the primary focus at presentation in 60.4% and 100% of their patients, respectively, while neurological presentation represented 27.1% and 33.8%, respectively, and immune dysregulation was 18.8% and 63.5%, respectively. On the other hand, Briggs et al. (2016) found that up to 50% of patients initially presented with immune dysregulation, followed by musculoskeletal symptoms (46.2%) and neurological symptoms (23.1%).

In our study, the same phenotype was seen in Patients 1 and 2 who were cousins signifying the absence of intrafamilial variabilities. Patient 3 had a milder phenotype and Patients 4 and 5 were the most neurologically affected ones. SPENCD is usually associated with variable degrees of intellectual disability as seen in Patients 1, 2 and 4 in addition to the brain imaging changes that were seen in our patients (Gelen et al. 2021). Intracranial calcification (ICC) is one of the silent features in SPENCD and was described in 62% of patients reported by Briggs et al. (2016) and was seen in 80% of our patients (4/5). ICC is usually bilateral in the basal ganglia and sometimes affects both frontal areas at the white and gray matter junction as seen in Patient 3. Therefore, SPENCD displays phenotypic overlap with the neuro-inflammatory interferonopathies such as AGS and patients with *ISG15* mutations. In fact, some of our patients were initially suspected to have AGS based on the clinical and neuro-imaging findings.

Immunological dysregulation is also a common feature in patients with SPENCD, and includes hepatitis, nephritis, rheumatic fever, hypogammaglobulinemia, recurrent pneumonia, disseminated herpes zoster, hemolytic anemia, autoimmune thrombocytopenia, pancytopenia, hypothyroidism, inflammatory myositis, systemic lupus erythematosus, juvenile rheumatoid arthritis, Moyamoya syndrome, Sjogren’s syndrome, Raynaud’s disease, scleroderma and vitiligo (Navarro et al. 2008; Briggs et al. 2011; Girschick et al. 2015; Bilginer et al. 2016; Kara et al. 2020). In this study, patients have not shown sufficient criteria for the diagnosis of autoimmune diseases at presentation. Nonetheless, immunological investigations revealed that three patients had deficiencies in cellular immunity (patients 1, 2 and 3) which were associated with elevated IgG and IgA in patients 1 and 2 and elevated IgG only in patient 2. Patient 4 had combined immunodeficiency along with low IgA. It is noteworthy that only patients 2 and 4 had a clinical presentation that might point to immune impairment. Immune dysregulation was suggested in patient 5 based on history of food allergy and eczema and demonstrated in immune parameters in the form of increased absolute counts of total T cells, T helper subset, B lymphocytes and NK cells along with increased IgA and total IgE. These findings agree with Roifman and Melamed (2003) who described SPENCDI in 4 patients and reported elevated IgG and decreased T cells in 3 of them. Girschick et al. (2015) also reported decreased T helper subset along with decreased B lymphocytes and NK cells. The variation in clinical signs and findings of immune dysregulation and immunodeficiency affirms the pleotropic nature of the disorder previously stated by Renella et al. (2006).

Literature review revealed that the skeletal phenotype was highly variable among the reported patients with SPENCD, with stature varying from normal range to -6.5SD. Exacerbation of the degree of short stature with age was evident in some patients. The type of short stature was also variable between short trunk short stature and short limbs’ short stature. An associated talipes, short distal phalanges, kyphosis, scoliosis, pectus carinatum, low bone mineral density and growth hormone (GH) deficiency were also reported. Surprisingly, some cases with minimal or nearly absent spondylometaphyseal changes and absent enchondroma-like lesions were reported despite they were considered the key diagnostic handles. Enchondroma-like lesions were absent in patients 1, 2 and 4 (Girschick et al. 2015; Briggs et al. 2016).

In general, response to GH therapy was controversial in patients with skeletal dysplasia. GH therapy showed a moderate effect on height gain in some patients, although it was claimed to be associated with side effects, such as femoral head necrosis, slipped capital femoral epiphysis, exostosis, and the progression of bone deformities, especially spinal deformities that necessitate cautious observation during therapy. Deformities could be related to the weak ligaments and matrix that support the muscles and bones and the effects of gravity on them. Nevertheless, further studies of genes that affect bone formation may clarify which skeletal dysplasia will properly respond to GH therapy (Kanazawa et al. 2003; Utsumi et al. 2017).

In SPENCD, the lack of tartrate-resistant acid phosphatase (TRAP) leads to an impairment of adhesion, migration and activation of osteoclasts and an incensement in phosphorylated osteopontin. Phosphorylated osteopontin is responsible for endochondral ossification and resorption of calcified cartilage matrix and primary spongiosa (Hayman et al. 1996; Janckila and Yam 2009; Behrens and Graham 2011; Lausch et al. 2011). GH stimulates longitudinal bone growth and stimulates resorption of cartilage through formation, differentiation and activation of osteoclast via osteoblasts. Up-regulation of insulin growth factor-I associated with GH therapy also supports osteoclastic activities. These effects of GH may counteract the negative effects on endochondral bone growth in patients with SPENCD (Mochizuki et al. 1992; Nishiyama et al. 1996).

**Fig. 5:**
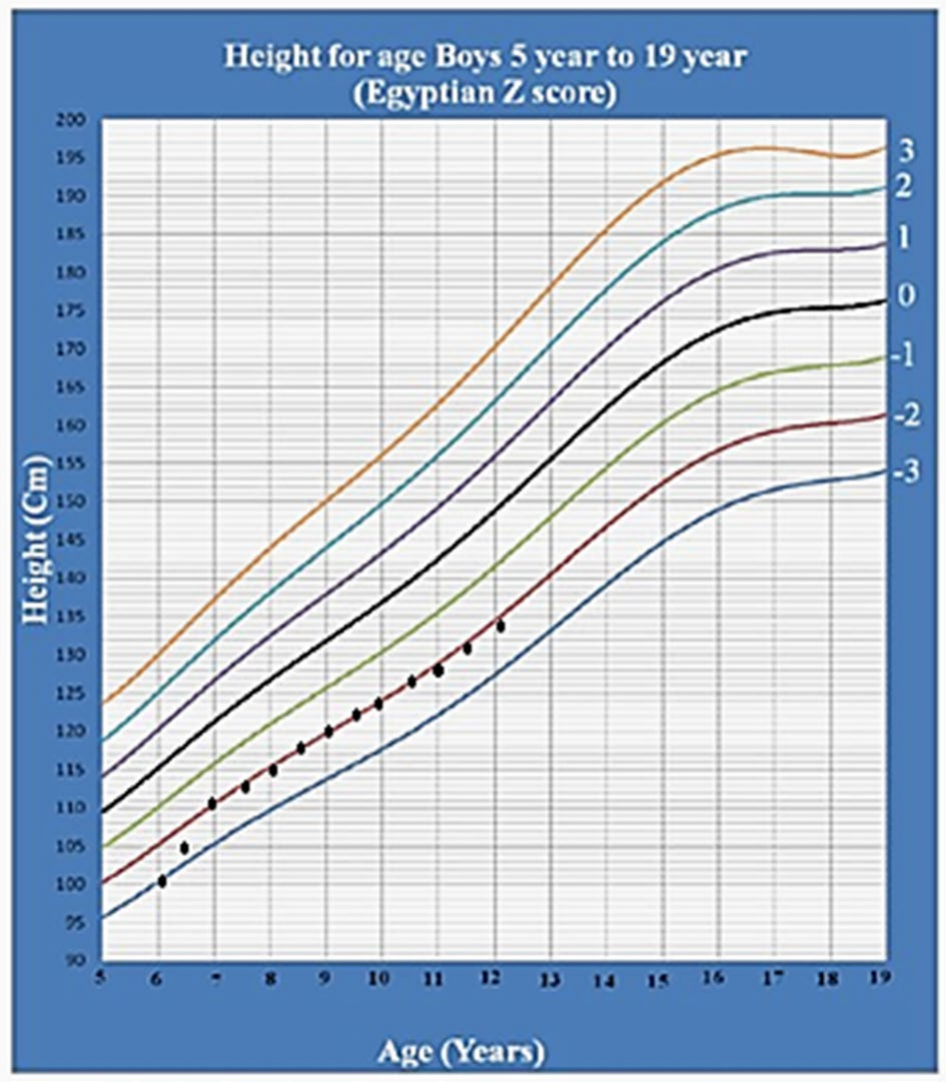
Height Z score chart for Patient 3 during GH therapy from age of 6 years to age of 12 years (the Egyptian height Z score chart by Shafie et al. 2020**)**

The therapeutic benefit of growth hormone in the management of short stature in SPENCD was clinically evaluated by Tuysuz et al. (2004) that described a patient who underwent GH therapy for 2 years from the age of 9 years, such that she achieved a normal height (153 cm: 3–10 centile). Moreover, Briggs et al. (2016) reported another patient with GH deficiency who showed a good response to GH therapy. Similarly, Utsumi et al. 2017 described a 3 year old boy with short stature who received GH therapy with a fair response too (his height improved from − 2.89 SD to − 1.04 SD over 3 years without accelerating the bone age). These findings come in line with our study as only one of our patients (Patient 3) received GH regularly for almost 6 years with height improvement, especially at the first 6 months from 100 cm (− 3.0 SD) at age of 6 years to 105.5 cm (− 2.41SD) at age of 6.5 years and 132 cm (− 2.35SD) at age of 12 years (Fig. 5). The mild kyphoscoliosic change that was noticed in Patient 3 at 12 years old might be related to the progressive nature of the disease itself or a side effect of the GH therapy. This clarifies the importance of close observation of the accelerated progression of skeletal deformities in treated patients.

To date, around 26 different *ACP5* variants have been identified in patients with SPENCD from various ethnic groups (Lausch et al. 2011; Girschick et al. 2015; Briggs et al. 2016; Bilginer et al. 2016; Sacri et al. 2017; Zhong et al. 2018; Kara et al. 2020**)**. These variants were 13 missense, 5 nonsense, 3 frameshift, 3 large intragenic deletions, 1 inframe deletion and 1 splice variant. Variants were distributed across the four coding exons of the gene without the presence of a single hot-spot or variants-cluster region. Generally, a few variants were recurrent in more than one family. The p.Gly109Arg was reported in three unrelated families from Iraq, Israel (Arab) and China (Briggs et al. 2016**)**. It is worthy to mention that the Iraqi family was of Jewish origin; therefore, the p.Gly109Arg might have a founder among Jewish Arabs. Similarly, the p.Lys52Thr and p.Gly215Arg variants were recurrent in two families each from Turkey and Africa, respectively (Briggs et al. 2016**)**.

In our study, each of the four families carried a unique *ACP5* variant. We identified two missense, one nonsense and one frameshift variant. The c.526C > T (p.Arg176Ter) and c.742dupC (p.Gln248ProfsTer3) are both predicted to result in nonsense-mediated decay of the protein. On the other hand, the two new missense variants affect highly conserved amino acid residues, which are absent in public genetic databases like gonmAD and different in-silico prediction tools supported their pathogenicity. Our variants raise the total number of *ACP5* causative variants to 29. Although the majority of reported *ACP5* variants were functionally null with absence of protein expression in the blood, no specific genotype–phenotype correlation was observed. In addition, a remarkable intra- and inter-familial phenotypic variability was observed in reported patients in the literature. This is partially in accordance with this study as we found a clear inter-familial variability but no intrafamilial variability noted in family 1. This might point to the presence of yet unidentified modifier genes or environmental factors that play role in this scenario.

In conclusion, we described five patients of SPENCD with severe neurological presentations that masked the associated mild skeletal and immunological signs. We recommend performing skeletal survey in all patients with spasticity and basal ganglia calcification even with the presence of minimal or absent joints deformities during clinical examination as it can help in early diagnosis of SPENCD patients. In addition, GH therapy showed a considerable outcome in the management of Patient 3; however, we recommend close observation to the possible accelerated skeletal deformities. Finally, we encourage more studies to evaluate the response of SPENCD patients to GH therapy.

## Supplementary Information

Below is the link to the electronic supplementary material.

Supplementary file1 (DOCX 24 KB)

## Data Availability

The data supporting the findings of this study are available with the corresponding author upon request.
