# Supplementary material for: Spondyloenchondrodysplasia in five new patients: identification of three novel ACP5 variants with variable neurological presentations
Source: Mol Genet Genomics. 2023 Apr 3;298(3):709–20. doi: 10.1007/s00438-023-02009-1 (PMC10133048; doi:10.1007/s00438-023-02009-1)
Supplement: Supplementary file 1 — Supplementary file1 (DOCX 24 KB) [file 438_2023_2009_MOESM1_ESM.docx]

Supplementary Table 1 The frequency in gnomAD and pathogenicity scores of the identified variants in our study

| **Variant** | **Allele Frequency in gnomAD** | **CADD** | **SIFT** | **Polyphen2** | **MutationTaster** | **Classification of variant according to ACMG** |
| --- | --- | --- | --- | --- | --- | --- |
| c.526C>T p.(Arg176Ter) | 0.000007990  (Two heterozygous carriers) | - | - | - | Disease causing  (Score 6) | Pathogenic |
| c.629C>T p.(Ser210Phe) | Absent | 29 | Deleterious (Score 0) | Probably damaging (Score 0.999) | Disease causing  (Score 155) | Variant of uncertain significance |
| c.742dupC p.(Gln248ProfsTer3) | Absent | - | - | - | Disease causing | Likely Pathogenic |
| c.775G>A p.(Gly259Arg) | Absent | 24.8 | Deleterious (Score 0) | Probably damaging (Score 0.999) | Disease causing  (Score 125) | Variant of uncertain significance |
